# Editorial Comment: Gastric Neobladders: surgical outcomes of 91 cases using different techniques

**DOI:** 10.1590/S1677-5538.IBJU.2017.0563.1

**Published:** 2018

**Affiliations:** 1 Department of Urology, University of Ulm, Germany

Many groups have addressed the issue of which gut segment is best suited for ONB reconstruction. All parts of the small and large intestine as well as the stomach have been intensively evaluatd. Ileum, which has been acknowleged as the optimum substitution material, has been mostly used and the ileal ONB has achieved statisfactory function.

The strength of the Brazilian paper from Porto Alegre (1) is its honesty of repording by the authors and reporting a significant eyperience of diffrerent gastric techniques, performed over a large time span, starting at a time point, when it was not clear, which intestinal segment would be best suited for bladder reconstruction. Furthermore it should be kept in mind that stomach has been largely endorsed for bladder augmentation in pediatric patients and stomach was highly recommended for ONB by renowned authors from leading institutions (2). From an operative standpoint, the gastric neobladder has several advantages. The gastric segment is easy to work with. Its lack of an intestinal anastomosis minimizes bowel manipulation. If desired, the thick muscular wall facilitates a tunneld ureteral anastomosis. The gastric segment easily reaches the urethra. The metabolic disturbanses from gastric bladder reconstruction are less severe and do not result in hyperchloremic metabolic acidosis and rarely result in clinically significant hyponatriemic, hypochloremic, alkalosis, which have been reported in patients with renal insufficency (3).

Most investigators have reported on a single type of continent urinary resorvoir. However, of particular interest is experience reported by others, non pioneering investigators and even more so, when they report on and compare different techniques. Unfortunately, this is rarely the case. Santucci et al. (4) reported the long term continence rates of ONBs and compared the urodynamik results in a series in which experienced surgeons performed a variety of different continent urinary reconstructions. They performed six different type of continent urinary reservoirs; the overtall numbers in each group were relatively small. However, their continence rates and urodynamic data were so remarkably different in the gastric and sigmoid neobladder groups that Santucci et al. are correct in believing their conclusions are justified despite the small patient numbers.

From a functional standpoint most authors report unsatisfactory urodynamic parameters for gastric neobladders. Compared with ileal neobladders, the gastric neobladders had a small capacity, lower compliance, and more frequent and stronger spontaneous contractions. Thus, the continence rates were appreciably lower than in ileal neobladders (33 % vs. 88 %) (4).

Many factors influence urinary continence including preservation of the autonomic innervation of the membraous urethra, avoidance of sphincter damage, a spheroidal reservoir with at least 4 segments ith opposing wall contractions, reservoir capacity between 400 and 500 ml with end fill pressure lower than urethral closing pressure at rest, absence of infected urine (causing reservoir wall contractions esulting in occasional sudden urine loss and increased mucus production), diabetes and patient age.

However, gastric neobladders result in inferior outcomes primarily related to the muscular nature oft he stomach.

In conclusion: Routine use of gastric neobladders in adults is not recommended. They may be appropriate especially as composites, in select cases such as renal failure or inadequate bowel length. The reasons for success in some patients and not in others are unknown
